# Open access, open education resources and open data in Uganda

**DOI:** 10.11604/pamj.2015.21.129.6325

**Published:** 2015-06-16

**Authors:** Ivana Di Salvo, Meggie Mwoka, Teddy Kwaga, Priscilla Aceng Rukundo, Dennis Ssesanga Ernest, Louis Aikoriogie Osaheni, Kasibante John, Kasirye Shafik, Agostinho Moreira de Sousa

**Affiliations:** 1 International Federation of Medical Students’ Associations; 2 University of Pavia, Italia; 3 University of Nairobi, Kenya; 4 Mbarara University of Science and Technology, Uganda; 5 International University of Health Sciences; 6 University of Benin, Benin; 7 Gulu University, Uganda; 8 Universidad do Porto, Portugal

**Keywords:** Open Access, medical journals, medical students

## Abstract

As a follow up to OpenCon 2014, International Federation of Medical Students’ Associations (IFMSA) students organized a 3 day workshop Open Access, Open Education Resources and Open Data in Kampala from 15-18 December 2014. One of the aims of the workshop was to engage the Open Access movement in Uganda which encompasses the scientific community, librarians, academia, researchers and students. The IFMSA students held the workshop with the support of: Consortium for Uganda University Libraries (CUUL), The Right to Research Coalition, Electronic Information for Libraries (EIFL), Makerere University, International Health Sciences University (IHSU), Pan African Medical Journal (PAMJ) and the Centre for Health Human Rights and Development (CEHURD). All these organizations are based or have offices in Kampala. The event culminated in a meeting with the Science and Technology Committee of Parliament of Uganda in order to receive the support of the Ugandan Members of Parliament and to make a concrete change for Open Access in the country.

## Introduction

Librarians, academia and researchers in Uganda have shown a continuous and resolute interest in the Open Access movement. Open Access journal publishing initiatives and support to Open Access repositories can prove it. The Makerere University Library, for example, was the first library in Uganda to set up an institutional repository called the Uganda Scholarly Digital Library (USDL). Launched as a science repository but later changed to cover other disciplines, USDL has a total of 1,600 full text articles, reports, posters, and other scholarly materials [1].

## Workshop report

The IFMSA participated in the OpenCon 2014 from 15-17 November 2014 in Washington DC, and the representatives (including Meggie Mwoka, Regional Coordinator for Africa, Ivana Di Salvo, Liaison Officer to Research and Medical Associations and Osman Aldiriri, Regional Assistant for SCORE - Standing Committee on Research Exchanges) came back with lot of enthusiasm and a willingness to share their knowledge and new ideas with the medical students. In order to follow up with the OpenCon 2014 a pre Africa Regional Meeting Open Access, Open Education Resources and Open Data was held in Kampala, Uganda from 15-18 December 2014. This pre- ARM workshop was organized within the series of ‘**Next Generation of Medical Researchers workshops**’. The previous two were pre- General Assembly workshops focusing on Research Integrity. To follow up with those pre-GAs, more workshops have been planned for the regional meeting as well and As Africa was the first region to host the meeting, we decided to focus on: a core part of research data; on educational resources fundamental for students and researchers; and, on research outputs and how to share them. Our main aim is to build capacities to launch and sustain Open Access, (Open Educational Resources (OER) and OD (Open Data) activities, projects and policies in the region, especially at national and local level. For this reason we decided to create a network in Uganda with local Universities and personalities based in the capital of Uganda, Kampala. The 10th African Regional Meeting was held in Kampala, Uganda under the theme Human Resources for Health: A foundation for Universal Health Coverage and was organized by the Federation of Uganda Medical Students Association (FUMSA). We received support from: Consortium for Uganda University Libraries (CUUL), The Right to Research Coalition [2], EIFL, Makerere University, International Health Sciences University(IHSU), Pan African Medical Journal(PAMJ) and the Centre for Health Human Rights and Development(CEHURD). In this way we promoted collaboration among students and professionals by encouraging local collaboration in Uganda for joint initiatives and campaigns, and cultivated this kind of approach within the IFMSA.

The students had the chance to meet and be trained by University Librarians from the International Health Sciences University and Makerere University Business School. The librarians are: Florence Mirembe who is also the Chairperson of the Consortium of Uganda University Libraries (CUUL) which promotes access to e-resources to support education and research within universities and other research institutions, and she is also the EIFL (Electronic Information for Libraries) country coordinator; Alison Kinengyere who is also the outgoing president of Association of African Health Librarians-Uganda Chapter and Hassan Segooba who is a librarian at the Makerere University Business School Library. In this way medical students can join their effort to promote Open Access. The participants also had the chance to get also the perspective of a medical journal editor thanks to the participation of Allan Mwesiga from the Editorial Office of the Pan African Medical Journal who shared the experience and achievements of an African Medical Journal as an Open Access journal. Under the guidance of Nsereko Ibrahim from the Centre for Health Human Rights and Development (CEHURD) participants learned more on promoting Open Access and on how to organize initiatives in Uganda, but also in other Universities with A case for CC licensing opportunities in Uganda.

## Conclusion

Through this event facilitated by Meggie, Agostinho and Mohamed, the students developed critical skills as well as advocacy and practical skills and had the opportunity to share their backgrounds, their project, how they have been involved in OA, OER and OD and analyse examples of successful projects with a view to planning on how to improve them or how to adapt them to their backgrounds. They are now looking forward to spreading awareness about Open Access. The event culminated in a meeting with the Science and Technology Committee of Parliament of Uganda, where the workshop participants presented a statement. There will be a follow up on this with the Ugandan Members of Parliament in order to receive their support and to make a concrete change in the country.
